# Detection and quantification of *Erysipelothrix rhusiopathiae* in blood from infected chickens – addressing challenges with detection of DNA from infectious agents in host species with nucleated red blood cells

**DOI:** 10.1099/jmm.0.001016

**Published:** 2019-06-07

**Authors:** Eva Wattrang, Victoria Jäderblom, Tomas Jinnerot, Helena Eriksson, Elisabeth Bagge, Maria Persson, Tina Sørensen Dalgaard, Robert Söderlund

**Affiliations:** ^1^ Department of Microbiology, National Veterinary Institute, SE-75189 Uppsala, Sweden; ^2^ Department of Animal Health and Antimicrobial Strategies, SE-75189 National Veterinary Institute, Uppsala, Sweden; ^3^ Department of Animal Science, Aarhus University, Blichers Allé 20, 8830 Tjele, Denmark

**Keywords:** *Erysipelothrix rhusiopathiae*, chicken, nucleated erythrocyte, blood, real-time polymerase chain reaction (PCR), droplet digital PCR

## Abstract

**Purpose:**

The present study aimed to establish pretreatment protocols as well as real-time and droplet digital polymerase chain reaction (PCR) methodologies to detect and quantify *Erysipelothrix rhusiopathiae* (ER) DNA in blood samples from infected chickens, as tools for routine diagnostics and monitoring of experimental infections. Chicken blood is a problematic matrix for PCR analysis because nucleated erythrocytes contribute large amounts of host DNA that inhibit amplification.

**Methodology:**

Using artificially spiked samples of fresh chicken blood, as well as blood samples from three experimental infection studies, the performance of pretreatment protocols, including choice of blood stabilization agent, centrifugation speeds and Ficoll gradient separation, was evaluated. The results were compared with those from traditional culture-based protocols combined with matrix-assisted laser desorption/ionization time-of-flight mass spectrometry (MALDI-TOF MS).

**Results/Key findings:**

Simple preparations producing cell-free samples performed well on artificial spike-in samples, providing high sensitivity. However, performance was poor in clinical samples or artificial samples where the bacteria were incubated for 4 h or more in fresh blood prior to DNA extraction. In these samples, a Ficoll separation protocol that creates samples rich in lymphocytes, monocytes and thrombocytes prior to DNA extraction was far more effective.

**Conclusions:**

Our results indicate that ER bacteria undergo rapid phagocytosis in chicken blood and that analysis of a blood fraction enriched for phagocytic cells is necessary for reliable detection and quantification. The presented results explain the poor performance of PCR detection reported in previously published experimental ER infection studies, and the proposed solutions are likely to have broader implications for PCR-based veterinary diagnostics in non-mammalian host species such as poultry and fish.

## Introduction

Blood samples are routinely used for a wide range of tests in both human and veterinary infectious disease diagnostics. While culture-dependent tests are still commonly used in bacteriology, techniques based on polymerase chain reaction (PCR) detection of pathogen DNA have been widely adopted for blood samples, as they are fast and cost-effective. Several components in blood, including haemoglobin, can inhibit PCR reactions [1], although in general this can easily be overcome with standard DNA extraction protocols. It has also been repeatedly observed that the presence of large quantities of host DNA or other irrelevant DNA in a sample can inhibit PCR [2, 3], as well as the related technique of droplet digital PCR (ddPCR) [4, 5]. The presence of host DNA is usually not an issue in the analysis of mammalian blood, since erythrocytes are by far the most common host cell type in these samples, and mammalian erythrocytes generally lack a nucleus and contain little or no DNA. Leukocytes are nucleated and also present in blood, but are far less numerous. In contrast, the erythrocytes of most non-mammalian vertebrates do contain a nucleus, and blood samples will thus contain very large quantities of host DNA. For example, the blood of both humans and chickens contains in the order of 10^12^ erythrocytes and 10^9^ leukocytes l^−1^ [6, 7], and the amount of host DNA is therefore in the order of a 1000-fold higher per volume unit in chicken blood compared to human blood. This poses a diagnostic challenge when analysing blood samples from chickens, as well as fish [8] and other non-mammalian vertebrates, with PCR. Unless host and pathogen cells can be separated in a preliminary stage, the inhibition can only be resolved by diluting the sample, resulting in an obvious loss of sensitivity. Metagenomic sequencing approaches to diagnostics hold great promise for the future beyond PCR [9], but they are even more sensitive to the presence of excessive host DNA.

In modern egg production, the disease erysipelas, caused by the Gram-positive bacterium *Erysipelothrix rhusiopathiae* (ER), is an increasing problem, especially in cage-free housing systems, including systems where hens have access to the outdoor environment, e.g. free range and organic production [10–16]. The disease manifests as outbreaks with high mortality (up to 60%) and egg production losses. The affected chickens display acute septicaemia and diagnosis is made through pathological findings in combination with isolation of ER from liver or spleen [15, 16]. Diagnostic culture of ER from necropsy samples or non-aseptically collected blood samples involves culture in selective media and takes 2–4 days, depending on the contamination of samples [16]; aseptically collected blood samples may shorten it to 2 days, but can be difficult to achieve in the field. Hence, it would be of great value if this process could be shortened by applying PCR methodology to detect ER DNA in chicken specimens. If such a protocol were applied directly to blood samples from affected flocks, a diagnosis could be made within 24 h. Therefore, the aim of the present study was to establish sensitive real-time PCR and ddPCR assays for the detection of ER DNA in chicken blood samples.

## Methods

### Bacterial strains

The ER strains 16-BKT031015 and 15-ALD003475, both derived from laying hens with clinical disease and *Erysipelothrix tonsillarum* strain CCUG31352, were stored at −70 °C and if not otherwise stated, bacteria were cultured on horse blood agar (National Veterinary Institute, Uppsala, Sweden) for 48 h at 37 °C before analysis.

### DNA extraction

DNA was extracted from bacterial preparations or different blood sample preparations (see below) by suspension in 100 µl water (Sigma-Aldrich), heating to 100 °C for 15 min and cooling at −20 °C for 10 min. Thereafter debris was removed by centrifugation at 10 000 ***g*** for 10 min and the supernatant as collected and stored at −70 °C until analysis.

### Quantitative real-time PCR assay

The primers and the ER-specific probe detecting the noncoding region downstream of the 5S rRNA coding region described earlier [17] were synthesized by Eurofins Genomics and used throughout this study. Real-time PCR reactions had a total volume of 15 µl containing 7.5 µl PerfeCTa qPCR ToughMix (Quantabio), 0.4 µM of each primer, 0.13 µM of probe and 2 µl of DNA samples.

Reactions were carried out in an ABI 7500 Fast Real-Time PCR system (Thermo Fisher Scientific) using MicroAmp Fast Optical 96-Well Reaction Plates (Thermo Fisher Scientific). The PCR cycling parameters were 3 min at 95 °C, followed by 45 cycles of 3 s at 95 °C and 30 s at 60 °C. The results were analysed with ABI 7500 Fast system software v 2.3 (Thermo Fisher Scientific). The threshold for each run was set at 0.2 to eliminate background noise before calculation of the cycle when the fluorescence threshold was reached (*C*_T_ value) for each positive sample. DNA from *E. tonsillarum* was used as negative DNA control. The bacterial DNA concentrations from each bacterial species, measured with the Qubit QuantIT HS assay, together with the predicted genome size for each species, were used to calculate the genome copy number µl^−1^. The ER DNA was diluted 1 : 10 in seven steps, from 2×10^6^ to 2 copies per reaction. Each dilution was analysed in triplicate to create a standard curve and confirm the expected sensitivity of the assay. Each sample from spike-in and experimental infection experiments was tested in duplicate and all DNA samples were analysed undiluted. In addition, samples from infected chickens in infection trial 3 were also analysed diluted 1 : 10 in water prior to addition to the PCR reaction. Each assay included a positive DNA control consisting of 2×10^2^ genome copies of the ER strain 16-BKT031015 and a negative no-template control (NTC) with the sample volume replaced by water.

### Droplet digital PCR assay

The same primers and probe as in the real-time PCR were used in the ddPCR. This assay was set up according to the Droplet Digital PCR Applications Guide (Bio-Rad; http://www.bio-rad.com/webroot/web/pdf/lsr/literature/Bulletin_6407.pdf). In brief, the ddPCR reactions had a total volume of 20 µl containing 10 µl ddPCR Supermix for Probes (Bio-Rad) 0.53 µM of each primer, 0.17 µM of probe and 2 µl of DNA samples. Droplets were generated in a QX100 Droplet Generator (Bio-Rad) and PCR reactions were carried out in a thermal cycler with PCR cycling parameters of 10 min at 95 °C followed by 40 cycles of 30 s at 94 °C and 60 s at 57.8 °C, followed by 10 min at 98 °C. After the amplification step, the droplets were analysed in a QX100 Droplet Reader (Bio-Rad) and the data were analysed using QuantaSoft software (v 1.5.38.1118). FAM fluorescent droplets were analysed in channel 1 and fluorescent and non-fluorescent droplets were separated with the threshold set at 4000 AU (Fig. 4). Samples with less than three positive droplets were considered to be negative. With this criterion and the dilution factors for analysing blood samples, the detection limit of the assay was approximately 1×10^4^ copies ml^−1^ blood.

### Preparation of ER-spiked chicken blood samples

The ER strain 15-ALD003475 was cultured for 48 h at 37 °C in meat broth (National Veterinary Institute). Subsequently, a 10-fold serial dilution of the bacterial suspension was performed and 100 µl volumes of each dilution were spread on agar plates and cultured for 48 h at 37 °C, and ER colonies were counted and colony-forming units (c.f.u.) ml^−1^ suspension were calculated. The bacterial suspension was mixed with EDTA-stabilized chicken blood from a good health blood donor layer flock (Håtunalab) to achieve concentrations of 10000, 1000 and 100 c.f.u. ml^−1^ blood, respectively, and blood samples were immediately prepared for DNA extraction according to protocol A (see below).

### Incubation of ER in chicken blood

A suspension of ER strain 15-ALD003475 cultured in meat broth was centrifuged at 10 000 ***g*** for 10 min and the pellet was resuspended in phosphate-buffered saline (PBS; pH 7.0, without Ca^2+^ and Mg^2^^+^). This bacterial suspension was added to either EDTA or heparin-stabilized chicken blood (Håtunalab) at ratios of 1 : 6 and 1 : 4, respectively. Each blood preparation was aliquoted into two samples that were incubated at 40 °C for either 4 or 16 h. After incubation, each sample was divided into three and prepared for DNA extraction according to protocols A, B or C, respectively (see below).

### Preparation of chicken blood samples for DNA extraction

#### Protocol A – ‘cell-free fraction’ (CFF)

Chicken blood samples were processed as previously described [18]. Cells were sedimented by centrifugation at 1700 ***g*** for 1 min and the supernatant was collected and centrifuged at 10 000 ***g*** for 10 min, after which it was discarded and the pellet was then stored at −80 °C until DNA extraction. In the case of the experimentally infected chickens in infection trial 1, 220 µl EDTA-stabilized blood was processed from each sample.

#### Protocol B – ‘slow-speed centrifugation’ (SSC)

Blood samples were diluted with an equal volume of PBS and centrifuged at 60 ***g*** for 15 min without brake in a swing-out rotor, and the plasma phase and the leukocyte layer above the red blood cells were collected and centrifuged at 10 000 ***g*** for 10 min, after which the supernatant was discarded and the pellet was then stored at −80 °C until DNA extraction.

#### Protocol C – ‘Ficoll separation’ (FS)

Blood samples were diluted with an equal volume of PBS and layered onto 1 ml Ficoll-Paque PLUS (GE Healthcare Life Sciences) and centrifuged at 400 ***g*** for 25 min without brake in a swing-out rotor. The interphase cells and all plasma above were collected and centrifuged at 10 000 ***g*** for 10 min, after which the supernatant was discarded and the pellet was then stored at −80 °C until DNA extraction. In the case of the experimentally infected chickens in infection trials 2 and 3, 220 µl EDTA-stabilized blood was processed from each sample.

### Experimental ER infection of chickens

The study comprised three separate infection trials with a total of seven groups of ER-infected SPF chickens (*n*=19–13 chickens/group) and one group (*n*=13) of uninfected chickens. The chickens, housing, trial designs and experimental procedures are described in detail in the Supplementary Material. In brief, the chickens were infected by intra-muscular injection of ER of the 15-ALD003475 strain on experimental day 0 and blood samples for serum were collected by jugular venipuncture from chickens on the indicated experimental days.

### Culture of ER inoculates for infection of chickens

The ER strain 15-ALD003475 was used for all infections of chickens. Details on the culture of the inoculates are provided in the Supplementary Material.

### Culture of ER in blood samples from infected chickens

#### Selective sodium azide/crystal violet broth

Immediately after blood collection, 10 µl unstabilized blood was transferred with a disposable cultivation loop to 5 ml selective sodium azide/crystal violet broth (National Veterinary Institute; containing 5 µg ml^−1^ crystal violet and 0.2 mg ml^−1^ sodium azide) and incubated for ER enrichment for 48 h at 37 °C. Then 10 μl of broth was spread on horse blood agar plates and incubated for 48 h at 37 °C. The identity of suspected ER colonies was subsequently verified by matrix-assisted laser desorption/ionization time-of-flight mass spectrometry (MALDI-TOF MS) on a Biotyper instrument (Bruker). This method was used on blood samples collected in infection trials 1 and 2.

### Direct culture

EDTA-stabilized blood samples were 10-fold diluted with PBS in three steps and 100 µl of undiluted blood and 1 : 10, 1 : 100 and 1 : 1000 dilutions, respectively, were added to horse blood agar plates and distributed evenly with plastic spreaders and incubated at 37 °C for 48 h before the counting of suspected ER colonies, based on morphology, and the identity of these colonies was subsequently verified by MALDI-TOF MS. This method was used for blood samples collected in infection trials 2 and 3.

## Results

### Evaluation of the ER real-time PCR assay

DNA from ER strain 16-BKT0310115 derived from a laying hen with clinical disease was used to determine the sensitivity of a previously published real-time PCR assay used for the detection of ER in tissue samples and oral swabs from pigs [17] and to calculate the amplification efficiency. The presence of ER was reliably detected at the level of 20 copies/reaction, with all replicates being positive, but it could not be reliably detected at the level of 2 copies/reaction, indicating that the detection limit is close to optimal in DNA samples from cultured bacteria. The amplification efficiency, *E*, was calculated from the slope of the standard curve as *E*=10^–1/slope^, to 98.5 %. The generated standard curve was further used to approximate the number of genome copies in clinical samples.

To evaluate the assay for inclusivity in terms of detecting genetically diverse types of ER, as well as exclusivity in terms of ability to distinguish ER from closely related bacteria, 10 diverse ER isolates representing the known clades of ER [19] and 3 *E. tonsillarum* isolates were selected. All ER isolates were positive, while all three *E. tonsillarum* isolates were negative.

Analysis of chicken blood samples ‘spiked’ with ER with blood samples prepared using protocol A, CFF, showed that the assay readily detected DNA from 10 000 to 100 c.f.u. ml^−1^ blood.

### Experimental ER infection of chickens – infection trial 1

**Fig. 1.**
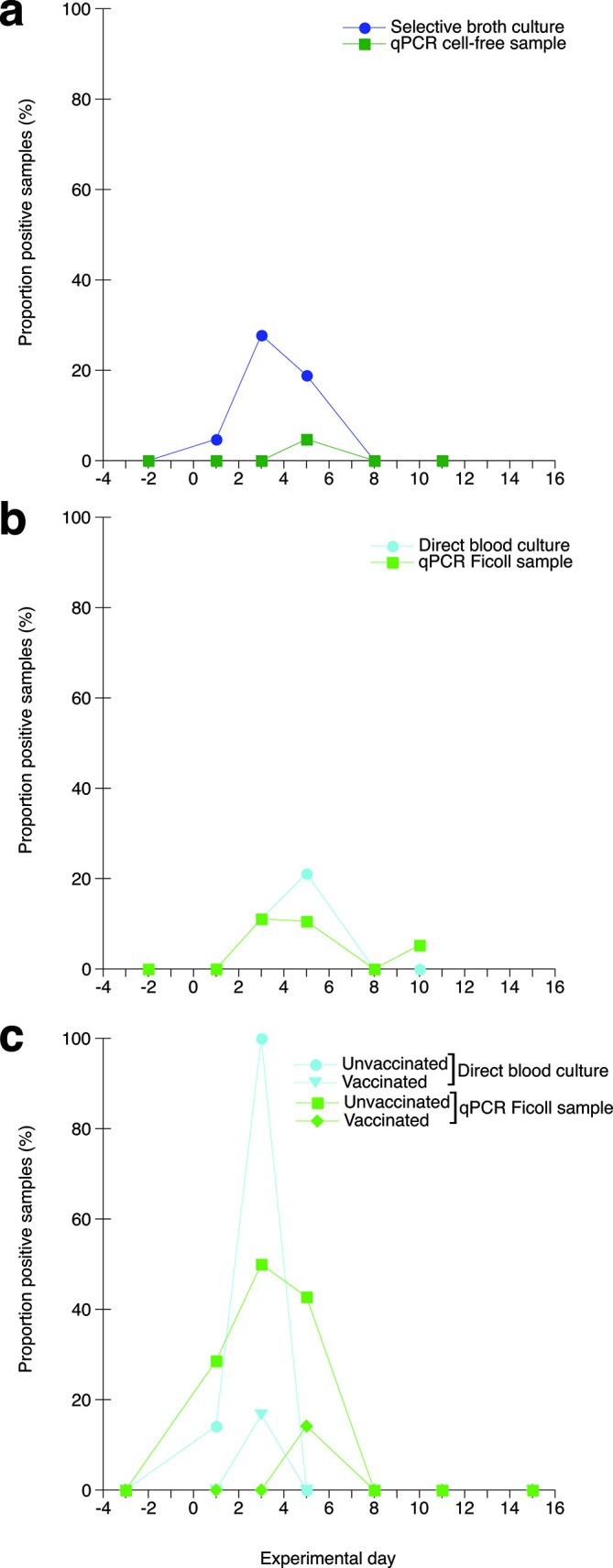
Proportions (%) of blood samples positive for growth of ER or positive for ER DNA quantified by real-time PCR, respectively, at the indicated days. Blood samples were collected from chickens experimentally infected with ER on day 0. (a) Infection trial 1: culture of ER was performed in selective sodium azide/crystal violet broth (dark blue circles) and real-time PCR was performed on samples prepared according to protocol A, cell-free (CFF; dark green squares). (b) Infection trial 2: culture of ER was performed directly on horse blood agar (light blue circles) and real-time PCR was performed on samples prepared according to protocol C, Ficoll (FS; light green squares). (c) Infection trial 3: culture of ER was performed directly on horse blood agar with blood from unvaccinated chickens (light blue circles) or chickens vaccinated against erysipelas (light blue triangles) and real-time PCR was performed on samples prepared according to protocol C, FS, with blood from unvaccinated chickens (light green squares) or chickens vaccinated against erysipelas (light green diamonds). For details see the Methods section.

In this trial, three groups of chickens, A, B and C, were infected with 0.5×10^5^, 0.5×10^6^ or 0.5×10^7^ c.f.u. ER/chicken, respectively (Table S1, available in the online version of this article). One chicken in group B showed mild signs of depression on day 4 after infection. No clinical signs of disease were observed among the other chickens during the experiment. Through culture of blood in sodium azide/crystal violet broth, growth of ER was detected in a total of 10 samples collected between days 1 and 5 after infection (Fig. 1a, Table S1). However, when using blood preparation protocol A, CFF, before DNA extraction, only one blood sample was positive for ER DNA (Fig. 1a, Table S1); 2.1×10^4^ genome copies ml^−1^ blood by real-time PCR and 6.8×10^3^ genome copies ml^−1^ blood by ddPCR, respectively. This sample was indeed also positive for ER growth.

Thus, it seemed that the real-time PCR was less sensitive in detecting ER DNA in samples from infected chickens than when using artificially spiked samples.

### Evaluation of different methods for blood preparation before DNA extraction

To test whether blood preparation methods that include leukocytes in the DNA extraction could enhance the yield of ER DNA from blood samples, live bacteria were incubated at 40 °C with freshly collected chicken blood to allow bacterial entry in to cells and/or bacterial uptake by leukocytes. After incubation, blood samples were prepared for DNA extraction in parallel using three different protocols: A – CFF; B – SSC; C – FS. Protocols B and C both included leukocytes, but protocol C resulted in the inclusion of a larger fraction of thrombocytes.

**Fig. 2.**
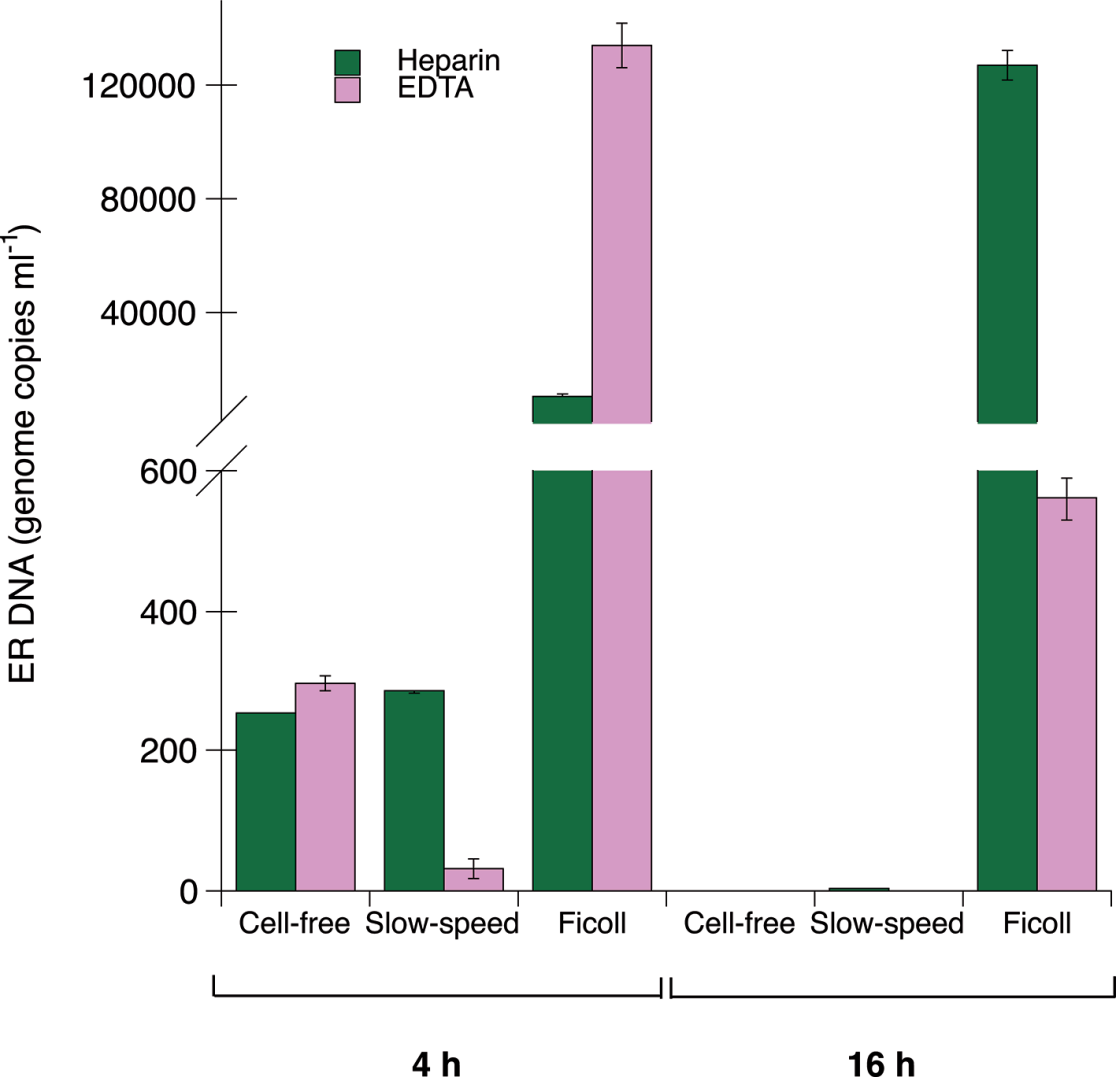
ER DNA quantified by real-time PCR in samples of heparin-stabilized (green bars) or EDTA-stabilized (light purple bars) chicken blood incubated at 40 °C for 4 or 16 h. After incubation, blood samples were prepared for DNA extraction in parallel using three different protocols: A, cell-free (CFF); B, slow-speed (SSC); C, Ficoll (FS); for details see the Methods section. The results are shown as the means of two technical replicates in the real-time PCR ±1 sd.

After 4 h of incubation, more than 30 times more ER DNA was detected in blood samples prepared using FS compared to CFF or SSC (Fig. 2). After 16 h of incubation, no or very small amounts of ER DNA were detected in blood samples prepared using CFF or SSC, while samples prepared with FS were strongly positive. There were some differences between the amounts of DNA detected in heparin- compared to EDTA-stabilized blood, but no clear influence of stabilizer was observed within this limited dataset.

Thus, it seems that ER may quickly enter/become phagocytosed in blood cells during *in vitro* mimicking of physiological conditions and that blood preparation by FS that includes leukocytes with a large proportion of thrombocytes increased the yield of ER DNA detected by PCR.

### Experimental ER infection of chickens – infection trial 2

**Fig. 3.**
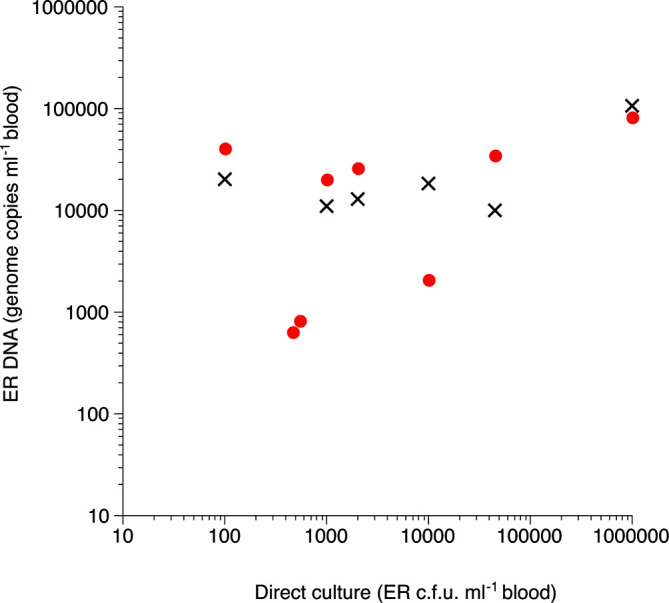
Correlation between c.f.u. of ER detected by culture and genome copies of ER DNA detected by real-time PCR (red filled circles) or by ddPCR (crosses) in blood from experimentally ER infected chickens in infection trials 2 and 3. Only samples that were deemed positive by both methods are shown and on those occasions where DNA samples were positive when both undiluted and in dilution, 1 : 10 results from the 1 : 10 dilution are shown. For comprehensive results, see Tables S3 and S5.

In this trial, two groups of chickens, D and E, were infected with 1.6×10^8^ or 1.6×10^6^ c.f.u. ER/chicken, respectively (Table S2). None of the chickens showed any clinical signs of disease during the experiment. Growth of ER was detected in a total of six samples collected on days 3 or 5 after infection by direct culture of blood (Fig. 1b, Tables S2 and S3). Growth of ER was only detected in four of these samples by culture of blood in sodium azide/crystal violet broth (Tables S2 and S3). Aliquots of all blood samples were also prepared according to protocol C, FS, prior to DNA extraction and analysed for ER DNA using real-time PCR. This analysis showed bacterial DNA in four of the six culture-positive samples on days 3 and 5 and in two samples that were negative for bacterial culture on day 10 (Fig. 1b, Tables S2 and S3). Samples that were positive for ER by either culture or real-time PCR were also analysed for ER DNA by ddPCR (Fig. 3, Table S3). In this infection trial, two of the samples that were positive by either culture or real-time PCR were also positive in the ddPCR. These two samples showed a good correlation between the amount of DNA detected by the two methods (real-time PCR vs ddPCR; 4.1 vs 2.1 and 2.6 vs 1.3, respectively, 10^4^ copies ml^−1^ blood). The approximate amounts of DNA detected in the samples that were only positive for ER DNA by real-time PCR were below the expected detection limit of the ddPCR.

Hence, the results indicated that the direct blood culture method was more sensitive than culture in selective medium and that the blood preparation protocol using FS increased the detection of ER DNA in culture-positive samples from infected chickens compared with the CFF protocol used in infection 1.

### Experimental ER infection of chickens – infection trial 3

**Fig. 4.**
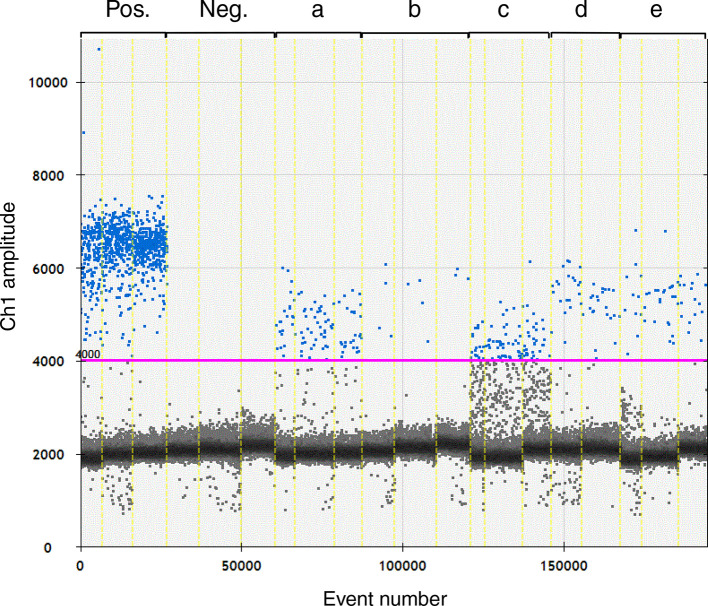
A selection of results from blood samples from ER-infected chickens (infection trial 3) analysed for bacterial DNA by ddPCR. Each lane (indicated by Pos., Neg. and a–eE) show the combined results from triplicate reactions of the same sample. The vertical axis shows the end-point probe fluorescence of each droplet PCR reaction in arbitrary units. Each individual event (droplet) evaluated after the completed PCR protocol is shown as a dot along the horizontal axis. Blue dots represent high-fluorescence positive events, i.e. droplets that contained one or more target DNA molecules. Grey dots represent negative events with low fluorescence levels, where no target DNA was present in the droplet. The purple line (4000 AU) represent the threshold level for positive events. Between 21570 and 33692 events were analysed for each of these samples. (a) Undiluted and (b) 1:10 dilution of DNA from chicken 25 day 3; (c) undiluted and (d) 1 : 10 dilution of DNA from chicken 38 day 3; (e) undiluted DNA from chicken 20 day 1; Pos., positive control with ER DNA; Neg., no-template control. For comprehensive results, see table S5.

This trial comprised three groups of chickens: group F (uninfected), group G (naïve infected) and group H (vaccinated and infected) (Table S4), and the chickens in groups G and H were infected with 0.5×10^10^ c.f.u. ER/chicken. One chicken in group G showed moderate signs of depression on days 2 to 4 after infection and did not gain weight during these days. This chicken also had the highest quantity of ER in blood, 10^6^ c.f.u. ml^−1^, when sampled on day 3 (Table S5). No clinical signs of disease were observed for the other chickens during the experiment. Growth of ER was detected in a total of eight samples collected during the experiment by direct culture of blood. On day 1 after infection, one chicken from group G was positive for ER growth, and on day 3, six from group G and one from group H were positive (Fig. 1c, Table S4). Among these positive samples, the highest c.f.u. ml^−1^ were observed in samples from group G (Table S5). For PCR analysis all blood samples were prepared according to protocol C, FS, before DNA extraction. When the real-time PCR was applied to undiluted DNA samples, two of the eight culture-positive samples were also positive for ER DNA (Tables S4 and S5). In addition, ER DNA was detected in one culture-negative sample on day 1 and in four culture-negative samples on day 5. When the real-time PCR was applied to DNA samples diluted 1 : 10, four of the eight culture-positive samples were also positive for ER DNA, while no culture-negative samples were positive for bacterial DNA (Tables S4 and S5). Samples that were positive for ER by either culture or real-time PCR were also analysed for ER DNA by ddPCR and a comparison of all three detection methods is shown in Table S5. Quantitative results from the direct blood culture and from the two different PCR detection methods are included in Fig. 3. As in infection trials 1 and 2, the ddPCR readily detected ER DNA where a large enough quantity of DNA was detected with the real-time PCR, e.g. one sample from day 1 (Fig. 4, lane e). However, two of the culture-positive samples that were negative when analysed undiluted in real-time PCR were positive for ER DNA when undiluted in the ddPCR, albeit showing slight (Fig. 4, lane a) or clear (Fig. 4 lane c) inhibition. These samples were also clearly positive in the ddPCR when tested diluted 1 : 10 (Fig. 4, lanes b and d).

Thus it seems that detection of ER DNA in blood may be inhibited in some undiluted DNA samples from infected chickens and that ddPCR seemed to be less sensitive to this inhibition.

## Discussion

The study objective to set up a methodology to detect and quantify ER DNA in blood samples from infected chickens was met with some challenges. In our first experimental ER infection of chickens only 1 of the 10 culture-positive blood samples was positive for ER DNA, even though validation of the real-time PCR showed that it detected DNA from low quantities of ER when the bacteria were mixed with chicken blood and the DNA was isolated immediately. Similar issues were evident in the study by Harada *et al*. [18], with a clear discrepancy between the high sensitivity of PCR when detecting DNA in spiked blood samples and the 100-fold lower detection of DNA in blood samples from infected chickens. The blood preparation protocol used in both studies eliminates the whole host cellular fraction before DNA extraction [18] and hence avoids the problem with excessive amounts of chicken DNA from the red blood cells, which should work well provided that all bacteria are extracellular. However, ER may survive and even proliferate in murine and porcine phagocytic cells (reviewed in [20]). Thus, we hypothesize that ER may persist in chicken phagocytic leukocytes. Some of the bacteria detected by culture may have been intracellular or adherent to cells and therefore lost during blood preparation for PCR, which would explain the discrepancy between culture and PCR results.

To test this hypothesis and evaluate if the blood sample preparation method prior to DNA extraction could be improved by the inclusion of leukocytes, we performed an *in vitro* pilot experiment. By culturing live ER in chicken whole blood, bacterial entry into cells and/or phagocytosis of bacteria by leukocytes could take place *in vitro* before two blood preparation protocols maintaining leukocytes, i.e. SSC and FS, were applied in addition to the CFF method. Our results showed that after 4 h of incubation higher amounts of ER DNA was detected in samples prepared by FS compared to SSC and CFF, respectively, and after 16 h of incubation, the samples prepared by FS remained clearly positive, while no or very small amounts of ER DNA were detected in the other samples. Ficoll gradient separation results in the enrichment of lymphocytes, monocytes and thrombocytes from chicken blood, while SSC gives a relatively pure lymphocyte population with only small amounts of thrombocytes [21]. In addition, the proportion of monocytes has been reported to be lower after SSC compared to FS [22]. Chicken blood contains three main populations of phagocytic leukocytes, namely heterophils, monocytes and thrombocytes [23–25]. Thus, it seems that the protocol that includes the most phagocytic cells also resulted in the highest amounts of ER DNA. Hence, these results indicate that ER may indeed be taken up by chicken phagocytic leukocytes. In this pilot experiment we used both EDTA- and heparin-stabilized blood. EDTA was suggested as a preferred anticoagulant, since heparin may inhibit PCR analysis [26], although EDTA has also been found to inhibit PCR reactions and it has also been suggested that the choice of anticoagulant for PCR analysis is less critical [27]. Moreover, the chelating properties of EDTA are considered to inhibit phagocytosis, e.g. by inhibiting calcium signalling [28]. However, it has also been show that *in vitro* phagocytosis can be observed in human whole blood cultures using EDTA-stabilized blood [29]. In the current limited dataset, both PCR reactions and phagocytosis seemed to work equally well in either anticoagulant, although the lower level of ER DNA recovered after FS of samples incubated for 16 h in EDTA- compared to heparin-stabilized blood may have been due to lower long-term viability of leukocytes in the former anticoagulant.

Ficoll separation was therefore applied to blood samples from ER-infected chickens in trials 2 and 3. In infection trial 2, a low proportion of infected chickens showed bacteria in the blood by culture, but with the exception of one sample with only 20 c.f.u. ml^−1^ blood, ER DNA was detected in all culture-positive samples. Thus, the real-time PCR showed the expected sensitivity when FS was performed prior to DNA extraction from blood samples. These results also indicated that ER may be intracellular in clinical samples. However, in infection trial 3, some of the culture-positive samples from day 3 post-infection were negative in the PCR when the undiluted DNA template was used. When the DNA template was diluted 1 : 10, some of these samples were found to be positive for ER DNA. Hence, it seemed that these DNA samples contained PCR-inhibiting substances, and the effect of these was overcome by dilution of the samples, although this regrettably reduced the sensitivity of the method. During this phase of the infection the blood leukocyte counts in the infected chickens increased by approximately sixfold (Wattrang et al., in preparation), which consequently would increase the amount of chicken DNA in the samples, and it seems likely that this DNA caused the observed inhibition. Thus, running diluted samples in parallel can clearly improve the overall detection rates. Because several blood parameters were analysed in parallel, we were restricted in the amount of blood (220 µl) that was available for DNA extraction in the present experiment, which in turn limited the sensitivity of the PCR assays. However, on a single occasion one may easily collect 2 ml blood from a mature chicken, and if this was solely used for DNA extraction the sensitivity of the PCR analysis could potentially be further improved.

Digital PCR, which is frequently implemented as ddPCR, as in the present study, is more laborious than real-time PCR and is therefore a less attractive option for diagnostics. However, ddPCR is generally considered to be a superior method for quantification and as a result of this provides valuable information, e.g. for following the progression of an infection. However, as previously observed by the authors and others, ddPCR can produce a small number of false-positive observations in any sample, which limits its ability to reliably detect and quantify very low target counts. When ddPCR was applied to samples from the infected chickens, ER DNA was readily detected in samples with large enough amounts of bacterial DNA for this assay and produced similar values for bacterial genome counts compared to real-time PCR, and also comparable c.f.u. values to those from traditional culture, offering a method for good quantification of DNA. Moreover, ddPCR also detected DNA in a few samples containing inhibitory substances that had not been detected in the real-time PCR without dilution, indicating that ddPCR had a slightly higher tolerance of inhibition.

In the present study, sensitive real-time PCR also detected ER DNA in some culture-negative samples. All of these samples were collected during the later stages of infection (day 10 in infection trial 2 and day 5 in infection trial 3), and most were from chickens that had tested positive for ER by culture at earlier sampling occasions. Thus, it seems likely that this ER DNA represents relic DNA [30] remaining in the host system after the bacteria have been killed, e.g. in the bloodstream or inside phagocytic cells.

In conclusion, in the present study we show that the combination of nucleated erythrocytes and rapid host phagocytosis of ER bacteria creates a need for appropriate pretreatment of chicken blood samples for the reliable detection and quantification of the pathogen. Combined culture and PCR-based methods provide optimal sensitivity, with culture being more likely to detect early infection, while samples are more likely to be PCR-positive in the later stages of ER infections. Some PCR inhibition by chicken DNA may occur during the acute phase of the infection, but at flock level, by including blood samples from a number of individuals, it should be possible to use this PCR methodology to rapidly diagnose the disease during an outbreak. We propose that the observed problems and suggested solutions have broader implications for the PCR detection of blood-borne intracellular pathogens – including, for example, viruses and protozoa, in addition to bacteria– in host species with nucleated erythrocytes.

## Supplementary Data

Supplementary material 1
