# Supplementary material for: Detection and quantification of Erysipelothrix rhusiopathiae in blood from infected chickens – addressing challenges with detection of DNA from infectious agents in host species with nucleated red blood cells
Source: J Med Microbiol. 2019 Jun 7;68(7):1003–11. doi: 10.1099/jmm.0.001016 (PMC6939158; doi:10.1099/jmm.0.001016)
Supplement: Supplementary material 1 [file jmm-68-1003-s001.pdf]

Description of experimental ER infections of chickens

Experiments were approved by the Uppsala regional Ethical Committee for Animal Experiments, permit no. C46/16. All chickens used were female Dekalb White layer hybrids purchased from a commercial hatchery and reared from day-old under SPF-conditions at the animal facilities at the National Veterinary Institute. Chickens were group housed in pens in rooms under negative pressure ventilation and after ER infection uninfected chickens and chickens infected with different bacterial doses were kept in separate rooms. Three different experimental ER infections were performed, described below. Prior to each infection chickens were weighed and allocated to groups to achieve an equal mean weight. Chickens were infected by intramuscular injection of 0.5 ml volume of inoculate at experimental day 0 of each infection trial. At blood sampling on experimental days 1, 3, 5, 8 and 11 (infection trial 3 only) the experimental groups were divided into halves and individual chickens were only sampled at every other occasion. Approximately 0.5 ml blood was drawn by needle and syringe under sterile conditions from the jugular vein of each chicken on the indicated days. Approx. 350 µl blood was transferred to sterile blood collection tubes with 1.0 mg EDTA-K_2 as additive (BD Microtainer MAP) and the remaining blood was added to sterile test tubes without additives.

Infection trial 1

This trial comprised of 39 chickens that were 22 days old at infection. The chickens were divided into three groups, n=13/group; chickens in group A, were infected with 0.5×10^5 cfu bacteria/chicken, group B, with 0.5×10^6 cfu and group C with 0.5×10^7 cfu. Blood samples were collected on experimental days -2, 1, 3, 5, 8 and 11.

Infection trial 2

This trial comprised of 37 chickens that were 26 days old at infection. The chickens were divided into two groups; chickens in group D, n=18 were infected with 1.6 x 10^8 cfu bacteria/chicken and group E, n=19 with 1.6 x 10^6 cfu. Blood samples were collected on experimental days -2, 1, 3, 5, 8 and 10.

Infection trial 3

This trial comprised of 39 chickens that were 30 days old at infection. The chickens were divided into three groups, n=13; chickens in group F were uninfected and injected with sterile broth, group G were infected with 0.5 x 10^{10} cfu bacteria/chicken and group H were vaccinated with a commercial erysipelas vaccine (Porcilis ERY Vet, MSD Animal Health) on day -13 and infected with 0.5 x 10^{10} cfu. Blood samples were collected on experimental days -3, 1, 3, 5, 8, 11 and 15.

Culture of ER inoculates for infection of chickens

The ER strain 15-ALD003475 was used for all infections of chickens. For infection trial 1 inoculation bacteria were cultured for 48 h at 37 °C in neat horse serum (Håtunalab). For infection trial 2 inoculation bacteria were cultured for 48 h at 37 °C in brain heart infusion broth (National Veterinary Institute). For infection trial 3 inoculation bacteria were cultured for 24 h at 37 °C on a shaker in tryptic soy broth (National

Veterinary Institute) supplemented with 0.1 % Tween 80, 0.1% D-glucose and 20 mg/L L-tryptofane. Numbers of ER in the inoculates was determined by a 10-fold serial dilution of inoculates were after 100 µl volumes of each dilution were spread on agar plates, cultured for 48 h at 37°C, ER colonies were counted and cfu per ml was calculated.

Supplementary table 1. Numbers of chickens positive for ER in blood either by culture or detection of bacterial DNA by real-time PCR after experimental ER infection, infection trial 1. Chickens were infected with the indicated number of bacteria (Dose) by intramuscular injection on experimental day 0 and blood was collected on the indicated experimental days.

			Experimental day					
Group	Dose (cfu)	Detection of ER	Day -2	Day 1	Day 3	Day 5	Day 8	Day 11
A	0.5×10^5	Selective medium culture[a]	0[c]/13[d]	0/7	1/6	1/7	0/6	0/13
		Real time PCR[b]	0/13	0/7	0/6	0/7	0/6	0/13
B	0.5×10^6	Selective medium culture	0/13	1/7	3/6	2/7	0/6	0/13
		Real time PCR	0/12[†]	0/7	0/6	1/7	0/6	0/13
C	0.5×10^7	Selective medium culture	0/13	0/7	0/6	1/7	0/6	0/13
		Real time PCR	0/10[†]	0/7	0/6	0/7	0/6	0/13

a – 10 µl blood was cultured for 48 h at 37 °C in selective sodium-azide crystal-violet broth before culture on horse blood agar (for details see section Materials and methods)

b – EDTA stabilised blood was prepared according to protocol A, CFF, before DNA extraction (for details see section Materials and methods)

c – number of positive samples

d – number of chickens sampled at respective occasion

† – Missing samples due to technical reasons

Supplementary table 2. Numbers of chickens positive for ER in blood either by culture or detection of bacterial DNA by real-time PCR after experimental ER infection, infection trial 2. Chickens were infected with the indicated number of bacteria (Dose) by intramuscular injection on experimental day 0 and blood was collected on the indicated experimental days.

			Experimental day					
Group	Dose (cfu)	Detection of ER	Day -2	Day 1	Day 3	Day 5	Day 8	Day 10
D	1.6×10^8	Direct culture[a]	0[d]/18[e]	0/9	2/9	1/9	0/9	0/18
		Selective medium culture[b]	0/18	0/9	2/9	0/9	0/9	0/18
		Real time PCR[c]	0/18	0/9	2/9	1/9	0/6	0/13
E	1.6×10^6	Direct culture	0/19	0/10	0/9	3/10	0/9	0/19
		Selective medium culture	0/19	0/10	0/9	2/10	0/9	0/19
		Real time PCR	0/19	0/10	0/9	1/10	0/8[†]	2/19

a – 100 µl EDTA stabilised blood was cultured on horse blood agar (for details see section Materials and methods)

b – 10 µl blood was cultured for 48 h at 37 °C in selective sodium-azide crystal-violet broth before culture on horse blood agar (for details see Materials and methods)

c – EDTA stabilised blood was prepared according to protocol C, FS, before DNA extraction (for details see section Materials and methods)

d – number of positive samples

e – number of chickens sampled at respective occasion

† – Missing samples due to technical reasons

Supplementary table 3. Comparison of results on detection of ER by direct culture, selective medium culture, real time PCR or ddPCR, respectively, in blood in samples positive for the bacterium either by culture or detection of bacterial DNA by real time PCR after experimental ER infection, infection trial 2. Chickens were infected by intramuscular injection on experimental day 0 and blood was collected on the indicated experimental days (Day).

Day	Group	Chicken #	Direct culture[a] (10^2 cfu/ml)	Selective medium culture[b] (+ or -)	Real time PCR[c] (10^2 copies/ml)	ddPCR[c] (10^2 copies/ml)
3	D	21	4.7	+	6.5	-
3	D	37	5.4	+	8.2	-
5	D	3	1.0	-	405.5	205.5
5	E	6	20.0	+	262.8	129.0
5	E	10	6.9	+	-	-
5	E	13	0.2	-	-	-
10	E	6	-	-	6.5	-
10	E	10	-	-	6.6	-

a – 100 µl EDTA stabilised blood was cultured on horse blood agar (for details see section Materials and methods)

b – 10 µl blood was cultured for 48 h at 37 °C in selective sodium-azide crystal-violet broth before culture on horse blood agar (for details see section Materials and methods)

c – EDTA stabilised blood was prepared according to protocol C, FS, before DNA extraction (for details see section Materials and methods)

+ – positive

- – negative

nt – not tested

Supplementary table 4. Numbers of chickens positive for ER in blood either by culture or detection of bacterial DNA by real time PCR after experimental ER infection, infection trial 3. Chickens were infected with 0.5 x 10^{10} cfu ER/chicken by intramuscular injection on experimental day 0 and blood was collected on the indicated experimental days.

			Experimental day						
Group	Treatment	Detection of ER	Day -3	Day 1	Day 3	Day 5	Day 8	Day 11	Day 15
F	Uninfected	Direct culture[a]	0[c]/13[d]	0/7	0/6	0/7	0/6	0/7	0/13
		Real time PCR[b]	0:0[e]/13	0:0/6†	0:0/6	0:0/7	0:0/6	0:0/7	0:0/13
G	Naïve	Direct culture	0:0/13	1/7	6/6	0/7	0/6	0/7	0/13
	infected	Real time PCR	0:0/13	2:1/7	1:3/6	3:0/7	0:0/6	0:0/7	0:0/13
H	Vaccinated	Direct culture	0:0/13	0/7	1/6	0/7	0/6	0/7	0/13
	infected	Real time PCR	0:0/10†	0/7	0:0/6	1:0/7	0:0/6	0:0/7	0:0/13

a – 100 µl EDTA stabilised blood was cultured on horse blood agar (for details see Materials and methods)

b – EDTA stabilised blood was prepared according to protocol C, FS, before DNA extraction (for details Materials and methods)

c – number of positive samples

d – number of chickens sampled at respective occasion

e – Real-time PCR on undiluted DNA samples : DNA samples diluted 1:10 (for details see Results)

† – Missing samples due to technical reasons

Supplementary table 5. Comparison of results on detection of ER by direct culture, real time PCR or ddPCR, respectively, in blood in samples positive for the bacterium either by culture or detection of bacterial DNA by real time PCR after experimental ER infection, infection trial 3. Chickens were infected by intramuscular injection on experimental day 0 and blood was collected on the indicated experimental days (Day).

				Real time PCR[b] (10^2 copies/ml)		ddPCR[c] (10^2 copies/ml)	
Day	Group	Chicken #	Direct culture[a] (10^2 cfu/ml)	Undiluted[c]	1:10[c]	Undiluted[c]	1:10[c]
1	G	17	-	6.1	-	-	-
1	G	20	450	344	189	99	-
3	G	22	5.0	-	-	-	-
3	G	23	1.0	-	-	-	-
3	G	25	100	-	21	185	174
3	G	35	10	158	202	111	-
3	G	38	10000	-	818	227	1080
3	G	39	3.0	-	-	-	-
3	H	37	0.3	-	-	-	-
5	G	14	-	38	-	-	-

5	G	17	-	25	-	-	-
5	G	20	-	16	-	-	-
5	H	1	-	9.2	-	-	-

a – 100 µl EDTA stabilised blood was cultured on horse blood agar (for details see Materials and methods)

b – EDTA stabilised blood was prepared according to protocol C, FS, before DNA extraction (for details see Materials and methods)

c – DNA samples were tested uniluted or diluted 1:10 in the real-time and ddPCRs

- – negative
